# Reform of the Medical Curriculum

**Published:** 1934

**Authors:** 


### REFORM OF THE MEDICAL CURRICULUM.

BY

A Medical Student.

---

### Foreword.

At the request of the Editorial Committee some suggestions for applying the Manchester proposals to the methods of clinical teaching at Bristol Medical School have been prepared by a senior student now engaged in clinical work. He has taken the opinions of his fellow-students, and the "suggestions," which are clearly and temperately expressed, may be regarded as representative of the present-day students' views. It should be realized that the medical students, when they reach the "clinical" part of the curriculum, are actually engaged in postgraduate study, except that they have not received a degree for pre-clinical study such as they would have obtained at Oxford or Cambridge.

---

The Manchester Medical School recently appointed a Clinical Commission to "prepare a report on the clinical instruction of students in Manchester and other medical schools with a view to submitting recommendations thereon to the Medical Board of the Royal Infirmary." This report has been published, and most of its conclusions and recommendations are worthy of consideration by the Medical Boards of many teaching hospitals.

The signatories to the report state that their suggestions are based upon two essential points. "First, that at least 75 per cent. of men engaged in the practice of medicine are general practitioners. Second, that . . . three factors are essential to the successful evolution of a competent general practitioner, namely theoretical knowledge, practical experience, and a sense of responsibility. The basis of training must be a 'learning by doing'; self-reliance and resourcefulness can never be developed in the student by lectures, demonstrations, and other passive educational procedures."

It will be convenient to examine the Manchester proposals in turn, suggesting any possible applications of them to the methods of teaching at present in use in the Bristol Medical School.

The Manchester proposals quoted here are placed in inverted commas.

### THE MANCHESTER PROPOSALS.

*Lectures.*—1. "Attendance at lectures should be voluntary. The system of taking a roll-call at lectures assumes immediately that the lecturer has nothing to offer of sufficient interest to ensure the voluntary attendance of students. . . . If attendance were optional, then the numbers present would be an index of their value in the eyes of the student."

2. "Systematic lectures should be drastically reduced in number. The chief value of systematic lectures is to serve as an introduction to the subject. With this object in view, brief introductory courses of systematic lectures in the elements and principles of the three major subjects might well be introduced."

The present series of Surgery Lectures given at Bristol admirably fulfils this purpose, and after attending them the student feels well equipped to deal with the commoner minor surgical conditions.

3. "Tutorial groups should be established to replace the systematic lecture courses, the groups consisting of as few students as possible; their work should invariably be clinical in nature."

4. "Correlation between all the branches of Clinical and Academic work is vitally necessary to prevent overlapping and unnecessary repetition. No attempt is made to correlate the teaching of Pharmacology, Pathology and Bacteriology with the final teaching in Medicine, Surgery and Obstetrics, nor do these three former subjects at any time attempt a specifically clinical approach in their teaching. At present all these subjects of the Final Degree Examination are kept in water-tight compartments, instead of being taught and preserved as the inseparable parts of the whole subject of Medicine, which they really are."

At present there is little or no clinical teaching in Therapeutics at Bristol Medical School, and Pathology is rarely mentioned or discussed in the wards.

In Surgery, and to a lesser extent in Obstetrics as well, there is an admirable series of Museum Classes. These are held once a week in the Museum of the Royal Infirmary: various diseases are discussed in systematic fashion, each condition being illustrated by pathological specimens, and by what is almost as valuable—the personal experiences of the demonstrator.

These classes could be extended. Certain honorary physicians and surgeons specialize in some branch of their work: these "specialists" might give a series of Museum Classes in their particular subject, illustrating them by specimens, personal experience, slides, and, most important of all, by showing cases. Two useful series of classes—one in Genitourinary Surgery, the other in Orthopædics—are given annually at Bristol on the lines suggested above.

A further suggestion is that attendance at these classes should be limited to the clerks or dressers of the demonstrator, and students who are undergoing the final eighteen months of their training.

The systematic lectures in Medicine, Surgery, and Obstetrics might well be replaced by intensive tutorial classes given during the various clerkships and dresserships. These, with the Museum Classes suggested above, would more adequately and satisfactorily cover the ground than do the present series of systematic academic lectures.

Applied Physiology needs more attention at Bristol. Only four or five demonstrations are given annually, and these to students who are about to take their examination in Anatomy and Physiology. A course of clinical demonstrations in this subject might profitably be given to students doing their medical clerkships.

With regard to Anatomy, a revision class in Pure Anatomy, given in the Dissecting Room, would be very useful to students in their final year.

Preventive Medicine is another subject rarely mentioned in the wards. Its practice should receive equal consideration with the treatment of the disease under discussion.

It is probably true to say that students are almost entirely self-taught in the art of interpreting skiagrams. A few demonstrations in this could profitably be included in classes given to clerks and dressers.

### *Lectures : Conclusions.*

1. " That clinical tutorials would be more effective in dealing with any subject which lends itself to this approach than any series of set lectures."

2. " That when lectures must be given, then the function of the lecturer should be : (*a*) To present a view of the subject as a whole ; (*b*) To state principles ; (*c*) To bring text-books up to date ; (*d*) To emphasize salient points in text-books by illustrations from the lecturer's own experience ; (*e*) To vitalize the subject."

"Lectures in Anæsthetics are, from this point of view, quite useless. Lectures in Applied Anatomy and Physiology could be completely replaced by lecture demonstrations and tutorials : the same applies to a large extent to the systematic lectures in Medicine, Surgery and Obstetrics. Acute medical, surgical and gynæcological conditions could be dealt with in a short series of lectures."

### THE WARDS.

The arrangements at Bristol, whereby students are allowed to examine patients, are quite satisfactory, although students occasionally find it difficult to obtain the help of nurses when examining female patients.

### ARRANGEMENTS FOR STUDENTS IN THE WARDS.

"Students are not entirely unnecessary so far as the running of the hospital is concerned, but they are actually a hindrance to the routine work carried on throughout the hospital."

The first part of this somewhat sweeping statement is certainly true of the Bristol Medical students : the second part is true of the "new student" in the wards or casualty department.

"This is keenly felt by the students and is, we think, the chief factor responsible for the lack of initiative and the general slackness which are so often found among clerks and dressers in the wards."

Most students arriving at the Royal Infirmary or General Hospital find themselves, in the words of the Manchester report :—

"Untutored and apparently unwanted, perpetually in someone else's way, and to all intents and purposes entirely superfluous."

In order to develop in the student practical and theoretical knowledge, with a sense of responsibility, the following scheme for clerkships and dresserships is suggested.

Clerks and dressers should be junior or senior, according to whether it is their first or second three months, respectively, or medical clerking or surgical dressing.

*Duties : Medical Clerks, Junior.*—Case notes and testing of urines, with instruction by the House Physician, and, later, some practice in the duties they will carry out as seniors.

*Seniors.*—Supervision of juniors ; prescribing of simple drugs and general treatment for own cases, under House Physician's supervision. Obtaining of specimens, and all minor clinical investigations, together with any other general routine treatment.

*Surgical Dressers.*—An equal or nearly equal number of senior and junior dressers should be attached to each Honorary Surgeon. Each junior to be under guidance and supervision of a senior, and the two to be allotted jointly the usual number of beds.

*Junior Dressers.*—Case notes and testing of urines, with instruction by House Surgeon and seniors.

*Senior Dressers.*—Routine treatment of own cases. Pre-operative treatment, certain (*e.g.* perineal, inguinal) dressings, bladder wash-outs, removal of stitches, tubes, etc., obtaining of specimens, writing up of and assisting at operations on own cases. Instruction of juniors in catheterization, venepuncture, bladder irrigation, etc.

The senior clerks and dressers should, so far as possible, be given practice in the prescribing for and treatment of patients.

*Theatre.*—The senior dressers should be second assistants to their Honorary Surgeons at operations on their own cases, or act as first assistants if the House Surgeon is administering the anæsthetic. They should be responsible for writing up the operation notes. The junior dressers should take charge of the instruments, leaving the swabs, needles, etc., in charge of the theatre nurse.

At present the Professor of Surgery demonstrates microscope slides of his operation cases once a week at the Infirmary. A useful extension of this would be for the Pathologist to demonstrate slides of all the week's operation cases.

### WARD ROUNDS.

These are often so well attended as to defeat their object by debarring many of the students from examining the cases and taking histories. It would be better if ward rounds were made by the Honorary, accompanied only by his houseman, clerks or dressers, and the sister.

### WARD CLASSES.

One Ward Class should be held each week in the clinical subjects by each Honorary Surgeon or Physician, attendance being restricted to final year students and the clerks or dressers of the Honorary.

One or two Ward Classes should also be held each week by the Registrar or housemen exclusively for the students working on the unit. The housemen should constantly supervise their students, giving them instruction in diagnosis, treatment, clinical investigation (ward and laboratory), and minor surgery. Further, students should have opportunities of observing and practising routine nursing treatment (enemata, packs, etc.).

It is most important that Ward Classes should not clash.

### SPECIAL SUBJECTS.

*Ear, Nose and Throat, Eyes, Skin.*

The teaching in these subjects is entirely satisfactory. The only complaint one can make is that the student has no opportunity, except in Dermatological Out-Patients, of carrying out treatment.

*Anæsthetics.*—At the Bristol Royal Infirmary a student acts as an anæsthetist's clerk for three months, during which time he personally gives as many anæsthetics as possible, the average number being about 100 cases. The institution of a course in Dental Anæsthetics would be beneficial.

*Post-mortem Department.*—Students should be allowed to assist at post-mortem examinations on their own cases.

*Obstetrics.*—Teaching in Midwifery and Gynæcology is admirable. The dressers are allowed to perform minor gynæcological operations, such as dilatation and curetage. The Midwifery Clerks, resident for six weeks each in rotation, are permitted to do versions, apply forceps, etc., under the supervision of the Resident Obstetrical Officer. But there is a danger that the Midwifery Department may come more and more to exist for the benefit of nurses taking the C.M.B course, thereby depriving the resident clerks of many cases.

*Children's Diseases.*—These should be more fully treated by the institution of a series of tutorial classes on the lines indicated for those in other subjects.

*Tuberculosis, Infectious Diseases* and *Vaccinations* are adequately treated in the Bristol curriculum.

*Psychology.*—A course in normal and abnormal Psychology should be given, correlated with the course in Mental Diseases.

*Orthopædics.*

"In Orthopædics the greatest need is for practical tuition in, and experience of, the treatment of fractures, application of splints and plaster, and so forth."

Most of the fracture cases arriving at a Bristol teaching hospital lose their teaching value. The student who sees them in the Casualty Department is usually unversed in the art of treatment, and told by the Casualty Officer to apply such and such a splint. He knows nothing of the mechanical or surgical principles involved, and is usually told nothing of the method of diagnosis and the X-ray appearances: a step in the direction of remedying this defect in the student's training has been the recent establishment of a "fracture clinic" at the Royal Infirmary.

*V.D. Clinics.*—More attention should be paid to this subject, and students given some opportunity of carrying out treatment.

### OUT-PATIENT DEPARTMENT.

"The Out-Patient Department of a teaching hospital is the nearest approach to the General Practitioner's Surgery."

At Bristol Medical School a student attends the Out-Patient Clinic of every Honorary for whom he works. This means that he is present in Out-Patients at least once, usually twice, a week for over two years. In his final year the student is at liberty to attend any clinic he wishes.

At first sight this system would appear to be almost perfect. In practice, however, some disadvantages become apparent. It means that there may be as many as eighteen students present at the Clinic of an Honorary who is known to teach well.

"It is pleasant neither for the patient nor the student to be in a constant state of siege. . . . It is easy, then, to understand that any student who examines more than two cases in a morning is extremely fortunate."

"The control of the attendance of students at these Out-Patients should be exercised by the Clinical Deans. If this suggestion is impracticable . . . students should be encouraged to visit Out-Patient Departments of other hospitals. . . . It should be made clear that Out-Patients are intended primarily for those in their final year or those who are acting as clerks or dressers to the Physician or Surgeon in charge."

"Finally, all histories of patients should be taken by students. The initial diagnoses, necessary investigations and treatment should be suggested by them."

### RESIDENCE OF STUDENTS.

"This is a matter of considerable import . . . a period of residence should be compulsory for students. It is our opinion that the present student, when qualified, may have neither the knowledge nor the experience of these things (medical and surgical emergencies) which a practitioner of medicine should possess before he attempts to enter into the practice of his profession. The purpose of compulsory residence is that the student should acquire this experience and this knowledge."

That Bristol Medical School is ahead of many other schools in the matter of students' residence is seen when comparing the practical knowledge of Bristol-trained medical men with that of men trained at some of the larger teaching hospitals. There is, however, still room for improvement.

The prospectus of the Bristol Medical School states that during their period of residence (one week each, in rotation, during the six months of surgical dressing) students attend to all casualties under the supervision of the house surgeons, and thus have more than ordinary opportunities of learning practical Surgery. But the resident surgical dresser is so pressed by duties —many unnecessary, and some entirely non-clinical— that he has little time to learn any surgery, even if any attempt were made to teach him, during his periods of residence.

The resident dresser is on duty all day and every day during his weeks of residence, when he has to perform his own clinical work and attend to his own patients in the wards. In addition, he is "on call" for Casualty and for any emergency ward duties. Yet from 9 a.m. to 4 p.m. both the Casualty and non-resident dressers are on duty, and available to attend to the patients allotted to them.

The resident dresser performs, among others, the following duties: Insert stitches, give anæsthetics, incise abscesses, and generally work under the direction of the Casualty Officer, prepare male patients for immediate operations, take all immediate operation cases to the theatre, and he may be allowed to assist at the operation, though this is a privilege now rarely given. After the operation he has to take the case back to a ward. Before midnight he has to take the day's collection of throat swabs, etc., from Casualty to the incubator in the Pathology Laboratory. During the night he is expected to perform catheterizations, bladder irrigations, etc., as may be required.

Without doubt this is excellent training for the arduous post of houseman, but porter's duties might be abolished, or if this is economically impossible, two dressers, senior and junior, might be "resident," with the less responsible duties allotted to the junior. This would enable the resident dresser to pay more attention to the clinical work and derive greater benefit during his period of residence.

### SUMMARY.

Briefly summarized, the suggested alterations to the teaching methods and clinical duties at Bristol Medical School are:—

1. The replacement, as far as possible, of systematic lectures by clinical tutorial groups.

2. The correlation of clinical and academic work.

3. The institution of a system of Junior and Senior clerkships and dresserships in Medicine and Surgery.

4. The limitation of attendance at Ward Classes and Out-Patient Clinics to clerks and dressers of the teacher and final year students.

5. The revision of the duties of the resident surgical dressers.

If any alterations in clinical teaching are contemplated at Bristol, the students would greatly appreciate the establishment of a committee, consisting of the Dean of the Faculty and Clinical Deans, together with some student representatives from the Royal Infirmary and General Hospital.

The students are very grateful for this opportunity of expressing their opinions on the subject of reform of the Bristol medical training.

